# Novel Population Specific Autosomal Copy Number Variation and Its Functional Analysis amongst Negritos from Peninsular Malaysia

**DOI:** 10.1371/journal.pone.0100371

**Published:** 2014-06-23

**Authors:** Siti Shuhada Mokhtar, Christian R. Marshall, Maude E. Phipps, Bhooma Thiruvahindrapuram, Anath C. Lionel, Stephen W. Scherer, Hoh Boon Peng

**Affiliations:** 1 Institute of Medical Molecular Biotechnology, Faculty of Medicine, Universiti Teknologi MARA, Sungai Buloh Campus, Selangor, Malaysia; 2 The Centre for Applied Genomics, The Hospital for Sick Children, Toronto, Ontario, Canada; 3 Jeffrey Cheah School of Medicine and Health Sciences, Monash University Sunway Campus, Selangor, Malaysia; 4 McLaughlin Centre and Department of Molecular Genetics, University of Toronto, Toronto, Ontario, Canada; Seoul National University College of Medicine, Republic of Korea

## Abstract

Copy number variation (CNV) has been recognized as a major contributor to human genome diversity. It plays an important role in determining phenotypes and has been associated with a number of common and complex diseases. However CNV data from diverse populations is still limited. Here we report the first investigation of CNV in the indigenous populations from Peninsular Malaysia. We genotyped 34 Negrito genomes from Peninsular Malaysia using the Affymetrix SNP 6.0 microarray and identified 48 putative novel CNVs, consisting of 24 gains and 24 losses, of which 5 were identified in at least 2 unrelated samples. These CNVs appear unique to the Negrito population and were absent in the DGV, HapMap3 and Singapore Genome Variation Project (SGVP) datasets. Analysis of gene ontology revealed that genes within these CNVs were enriched in the immune system (GO:0002376), response to stimulus mechanisms (GO:0050896), the metabolic pathways (GO:0001852), as well as regulation of transcription (GO:0006355). Copy number gains in CNV regions (CNVRs) enriched with genes were significantly higher than the losses (P value <0.001). In view of the small population size, relative isolation and semi-nomadic lifestyles of this community, we speculate that these CNVs may be attributed to recent local adaptation of Negritos from Peninsular Malaysia.

## Introduction

Southeast Asia is believed to be one of the earliest regions of *Homo* genus habitation recorded outside Africa. This may have occurred nearly 2 million years ago, following the arrival of the ancient Javanian, known as *Homo erectus* [1]. The Negrito people are believed to be direct descendants of humans who arrived in Peninsular Malaysia more than 60,000 years ago [2]–[4]. Ancestral *Homo sapiens* who originated from Africa [5] migrated into Asia along the coastal route [6]. The Negritos from Peninsular Malaysia are of Austroasiatic origin [7] and thought to be related to the Philippine Aeta and Andaman Islanders as well as the Melanesians, Tasmanians, and certain tropical Australian rainforest foragers based on superficial anatomical features and foraging lifestyles [8]. In Malaysia, Negritos are divided into six tribes based on linguistics, socio-cultural practices, and geographical region inhabited namely, Bateq, Mendriq, Jehai, Kensiu, Lanoh and Kintak, numbering approximately 0.15% of the total population [9]. Studies on various genetic markers including autosomal microsatellite markers and mitochondrial DNA suggest that these tribes are genetically similar and may have experienced high levels of genetic drift [8], [10], [11]. It is believed that they may have adapted to the environmental changes throughout the centuries to cope with limited food resources and the tropical rainforest environment. Currently, the number of Negritos is dwindling rapidly as Malaysia becomes more developed and forests are cleared. Characterizing the genetic variation of the isolated populations such as Negritos provides valuable information to the gene mapping of complex diseases [12]. Thus it is crucial to unveil their genetic makeup in order to better understand how genetic variation contributes to the well-being and health of human populations especially in the Southeast Asian region.

Copy number variations (CNV) typically range from 1 kb to several megabases in size [13] and are acknowledged as a major contributor to genetic diversity. This variability plays an important role to determine phenotypes such as physical features and conferring susceptibility to a number of common and complex diseases including HIV, psoriasis, and a number of neuropsychiatric diseases [14]–[17]. This occurs via potentially altering gene expression levels and influencing the gene dosage [18], [19]. They account for a significant proportion of the genome [13], [20], are highly variable, and often harbor regions with genes sensitive to the environmental stimulation such as those involved in immunity, metabolism, olfactory receptors [21]–[23]. Due to their non-random distribution across the genome, it is believed this phenomenon may have trended towards selection bias [21], [24].

Most genetic diversity data in indigenous populations have been based on single nucleotide polymorphisms (SNP)/single nucleotide variations (SNV) [6], [25], [26] and maternal lineage mitochondrial DNA [4], [8], except for a handful of studies [27]–[33]. To date, CNVs in indigenous populations of Peninsular Malaysia have not been reported. As a complement to the existing SNP data, we explored the first CNV map of Negrito individuals from Peninsular Malaysia and report the distribution of novel and population-specific CNVs. Our findings may be able to provide fundamental insights to the genetic architecture of the Negritos which can be translated to aid biomedical and evolutionary investigations.

## Materials and Methods

### Sample Recruitment

This study was reviewed and approved by the Research and Ethics Committee of Universiti Teknologi MARA [Ref no: 600-RMI (5/1/6)], and Department of Orang Asli Development (Jabatan Kemajuan Orang Asli Malaysia, JAKOA) [Ref no: JHEOA.PP.30.052.Jld 5(17)]. Prior to sample collection, the headman of the tribe and/or the community members were first consulted in a customary courtesy visit and their consent were obtained. During sampling, all participants were interviewed, and informed and written consent were obtained. Process of interview and informed written consent was conducted in Malay language and witnessed by the officer from JAKOA. Only Negrito participants 18 years who gave consent were selected. We collected 10 ml of peripheral blood from 34 unrelated individuals (17 males and 17 females) after obtaining informed consent. The samples consisted of both males and females from sub-tribes Jahai, Bateq, Mendriq and Kensiu. DNA was extracted from using Qiagen Blood Extraction Kit (Qiagen, Hilden, Germany) according to the manufacturer's protocol.

### Microarray Genotyping

Genotyping was performed using the Affymetrix SNP6.0 Array platform according to the manufacturer's instructions. Briefly, 250 ng of genomic DNA was digested and ligated. The ligated products were then PCR amplified. Amplicons were electrophoresed, purified and quantified to ensure that the samples passed quality control (QC) measures before further experiment. The products were then fragmented, hybridized onto the Affymetrix SNP6.0 chips and stained. Chips were scanned and raw data was generated using Affymetrix Genotyping Console Software (GTC) version 3.0.2 with default settings.

### Copy Number Variation Analysis and Validation

CNVs were called independently using three algorithms, Affymetrix GTC, Birdsuite and iPattern (TCAG) as described previously [35]. We applied stringent filtering criteria such that CNV had to be a minimum of 1 kb and span 5 consecutive probes, and be detected by at least 2 out of the 3 algorithms. In addition we excluded CNVs that were on the X and Y-chromosomes, or approximately 300 kb adjacent to the centromeres and telomeres. To define a set of rare CNVs we excluded known polymorphic loci (ie. Copy number polymorphism, CNP, targeted by the array) and those CNVs with more than 50% reciprocal overlap with those reported in DGV.

The filtered CNV calls were then compared with the HapMap3 dataset and subsequently the Singapore Genome Variation Project (SGVP) (http://www.statgen.nus.edu.sg/~SGVP/), to further identify CNVs unique to the Peninsular Malaysia Negritos. We defined a CNV as putative novel and unique to Negritos (denoted as population-specific CNVs) when it is not present in any of the HapMap3 and SGVP samples (defined as <50% reciprocal overlap with HapMap3 and SGVP CNVs).

Annotated CNVs unique to the Negrito samples studied with underlying genes were validated with qPCR SyBr Green assay as previously described [34]. A total of 50 ng (10 ng/µl) genomic DNA was amplified in a reaction mixture containing 12.5 µl iQ Sybr Green Supermix (Biorad), 1 µl (7 µM/µl) of respective forward and reverse primers, and top up to total volume of 25 µl with ddH_2_O. Cycling conditions were 95°C for 3 min, and then 40 cycles of 95°C for 30 s, followed by respective annealing temperatures of each locus for 15 s and 72°C for 30 s.

Melting curve was performed to check for specificity of the assay. Efficiency of the assay was observed by the generation of standard curve by created a serial of five-fold dilutions of a top standard of 50 ng/µl to 0.08 ng/µl (10 ng to 0.016 ng) of a single genomic DNA sample. All reactions were run in triplicate, except a few when the genomic DNA was insufficient, were run in duplicate. Normalization to the control gene Forkhead Box P2 (FOXP2) (primers 5'-TGACATGCCAGCTTATCTGTTT-3' and 5'-GAGAAAAGCAATTTTCACAGTCC-3') was used to give an estimate of copy number. The reproducibility of the qRT-PCR assay for each sample was calculated by estimating the within-sample variation measured through the coefficient of variation (C.V. %  = 100*[standard deviation]/mean). Copy number of the target sequence in each test sample is determined by using comparative CT (2-ΔΔCT).

Eight out of 12 (66.7%) CNVs were true positive (8 out of 9 were CNVs >10 kb in length). However, all 3 CNV less than 10 kb failed to validate. Considering the low replication rate, we removed the CNVs sized <10 kb from further analysis. The primer sequences and the copy number amplified for the candidate CNVs is listed in Table 1.

**Table 1:** Candidate genes primer sequences and copy number amplified in SyBr Green qPCR assay.

Locus name	CNV size spanned (bp)	Primer sequence	Expected amplicon size (bp)	Annealing temp (°C)	Copy number
ADH7	153,593	Forward: gaaggcacaagctgctgttatReverse: catcctgtctttgtcttggatct	99	59.6°C	3(2.80, 0.108)
CSMD1	301,535	Forward: actctgaacggtgtcctggttt Reverse: ttcctaagctgcaaaggtgtg	92	62.2°C	3(3.11, 0.062)
SH2D4B	15,464	Forward: atgttctatgctgtggtggatg Reverse: acgaactttgtcagaaacgtga	101	59.9°C	1(0.45, 0.042)
NPAS3	25,484	Forward: ctgttggcttagaggctgagatReverse: agcccttgagatgattcctaca	109	60°C	1(1.32, 0.65)
WDR4	165,544	Forward: acaggtttgtgagccgtatctcReverse: tcaagaatccagaggtgagtga	106	60°C	2(2.10, 0.14)
LRRC30	9,547	Forward: cttgcacgtgggctcgaatcReverse: ggatgttgttgccctctgcg	95	66.3°C	-
TNFRSF1B	83,214	Forward: cattaggagatgtgtggtcctgReverse: aacagtatgtcccgttctgtctc	90	59.6°C	3(3.09, 0.008)
PRIMER 1	36,283	Forward: acagaacctaagcggaaatcctReverse: aactggaagcaagatgctgact	107	64.0°C	3(3.40, 0.08)
PRIMER 2	65,481	Forward: ccctgaagcgtgagtctctaat Reverse: tgataacacctctgcacattcc	89	63.5°C	3(2.50, 0.12)
PRIMER 3	42,399	Forward: ggtcttcagtttgtgcttcagat Reverse: catcacttcctagcgccttc	80	63.4°C	3(2.90, 0.07)
PRIMER 4	63,260	Forward: tcctaaagtttccgcaggagReverse: ctcacttcactggtgtcaggtt	99	63.2°C	1(1.14, 0.32)
QCNV2	9,812	Forward: caggcaagttcatatgttccaReverse: agaggaatgccagatagagcag	113	63.6°C	3(2.90, 0.11)
QCNV4	4,021	Forward: acttggtaaattgtgttgaReverse: tgtcagtcctgcattt	104	52.4°C	2(2.20, 0.17)

WDR4 and QCNV4 showed copy number normal and therefore considered as false positive. QCNV2 was detected as a CN gain by microarray, inconsistent with the qPCR validation, therefore considered as false positive. Parentheses, unrounded copy number values calculated using the relative quantification, standard deviation.

The microarray dataset has been submitted to NCBI dbGaP. The accession number assigned is: phs000664.v1.p1.

### Gene Ontology Analysis

We submitted the annotated genes list underlying the Negrito-specific CNVs observed to PANTHER (Protein ANalysis THrough Evolutionary Relationships) (http://www.pantherdb.org/) and DAVID (the Database for Annotation, Visualization and Integrated Discovery, version 6.7) (http://david.abcc.ncifcrf.gov/summary.jsp).

## Results

### General Characteristics of CNV and CNVR

**Figure 1:**
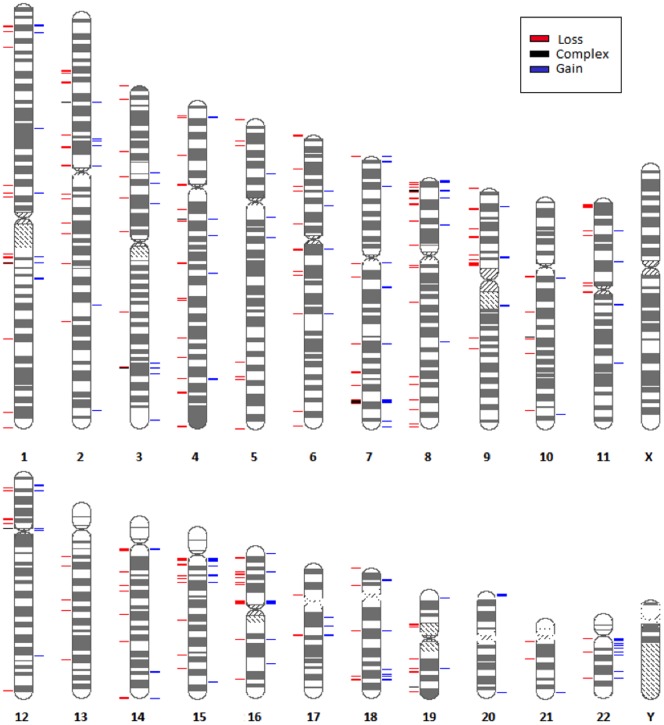
CNVR map of Negrito samples. The ideogram summarizes the distribution of CNVRs on each human chromosome. The red indicates copy number loss, the blue indicates copy number gain while the green indicates multi-allelic loci.

**Figure 2:**
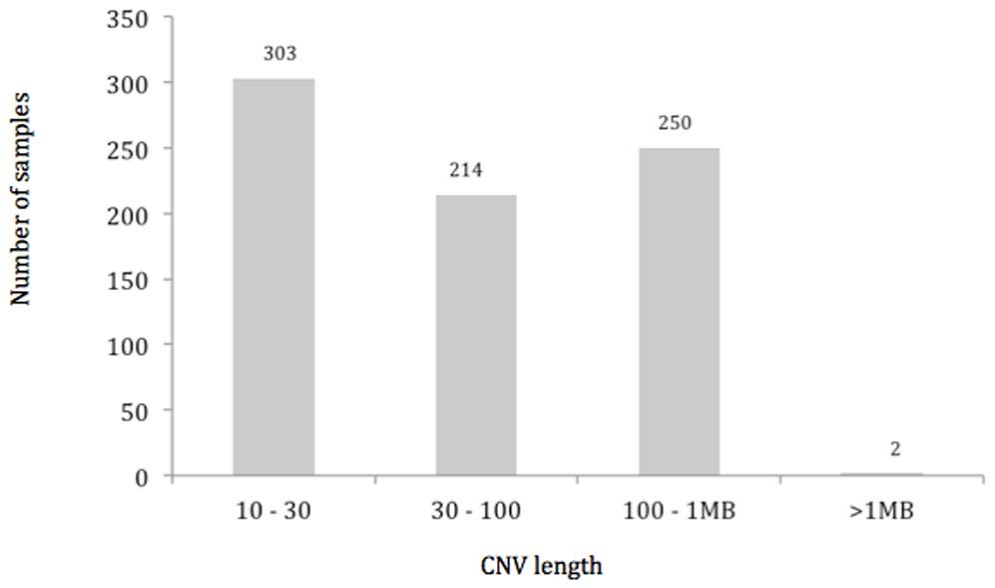
Length distribution of the CNVs in Negrito from Peninsular Malaysia.

We identified 1,333 autosomal CNVs from Genotyping Console (Affymetrix), with an average 39.2 CNVs per genome, whilst the total number of CNVs being called by Birdsuite and iPattern were 2,636 and 3,692, respectively (mean number of calls per genome 77.5 and 108.6 respectively, Table 2). After applying stringent filtering criteria, 1,111 overlapping CNVs were successfully merged, with an average 32.7 CNVs per genome (CNV call per genome ranged from 19–54), corresponding 105,909,572 bp of the total autosomal genome (Figure 1). These corresponded to 263 CNVRs comprising of 161 losses, 94 gains and 8 multi-allelic sites. Figure 2 shows the length distribution of CNVRs in this study.

**Table 2:** General characteristics of CNV and CNVR among 34 Negrito genomes from Peninsular Malaysia.

	GTC	Birdsuite	iPattern	Merged*
**Total CNV count:**				
Gain	530	735	1,430	330
Loss	803	1,901	2,262	781
Complex CNV			40	
Total	1,333	2,636	3,692	1,111
**Average number per genome:**				
Gain	15.5	21.6	42.0	9.7
Loss	23.6	55.9	66.5	23.0
Total	39.2	77.5	108.6	32.7
**Size (bp):**				
Min	1,000	1,019	1,010	1,134
Max	1,768,000	985,807	1,033,784	1,033,785

*Merged: stringent CNV calls by at least 2 out of 3 algorithms applied.

### Comparison of Common CNVs

**Figure 3:**
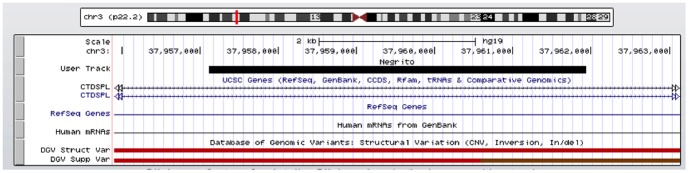
UCSC Genome Browser view of CNV on chromosome 3p22.2. Figure produced by custom tracks listing CNV call of Negrito and uploaded to http://genome.ucsc.edu.

We first compared the diversity of common CNVs with the HapMap3 populations derived from 10 populations (consisting 1,072 samples). A set of CNVs that showed significant differences of allele frequencies are listed in Table 3. Notably, CNV losses at chromosome 3p22.2 (37,957,108–37,961,932) were observed in 56% of the Negrito samples in this study as compared to the rest of the HapMap3 populations (Table 3; Figure 3). The gene CTDSPL involved in this CNV was found to be associated with prostate cancer (https://www.genome.gov/26525384). The CNV in chromosome 15q13.3 was another region of interest. Frequency of this CNV was found to be higher (0.44) as compared to the HapMap3 samples (ranging from 0.09–0.21). The gene CHRNA7 involved in this CNV was found to be associated with schizophrenia and epilepsy [35], [36].

**Table 3:** Common CNV with significant difference in allele frequencies compare to the HapMap3 dataset.

CNVs	Chr	Start	End	CNV Frequencies										
				NEG	ASW	CEU	CHB	CHD	GIH	JPT	LWK	MEX	TSI	YRI
1.	2	40,780,879	40,803,110	0.21	0.00	5.6×10^3^	0.06	0.02	0.00	0.00	0.00	0.00	0.00	0.00
2.	2	52,605,074	52,635,046	0.88	0.17	0.53	0.80	0.80	0.70	0.86	0.04	0.44	0.49	0.09
3.	3	37,957,108	37,961,932	0.56	0.07	0.11	0.15	0.16	0.21	0.03	0.01	0.11	0.27	0.03
4.	4	78,495,579	78,500,367	0.12	0.00	0.00	0.03	0.07	0.00	0.03	0.00	0.01	0.00	0.01
5.	6	202,353	326,149	0.03	0.15	0.29	0.26	0.37	0.16	0.29	0.17	0.27	0.30	0.09
6.	15	32,487,975	32,617,680	0.44	0.21	0.09	0.16	0.17	0.18	0.22	0.10	0.15	0.17	0.21
7.	16	14,897,364	15,016,088	0.09	0.30	0.24	0.42	0.35	0.30	0.36	0.33	0.31	0.21	0.22
8.	17	41,750,187	42,107,479	0.18	0.00	0.00	0.01	0.02	0.00	0.03	0.00	0.02	0.00	0.00

### Population Specific CNVs

**Figure 4:**
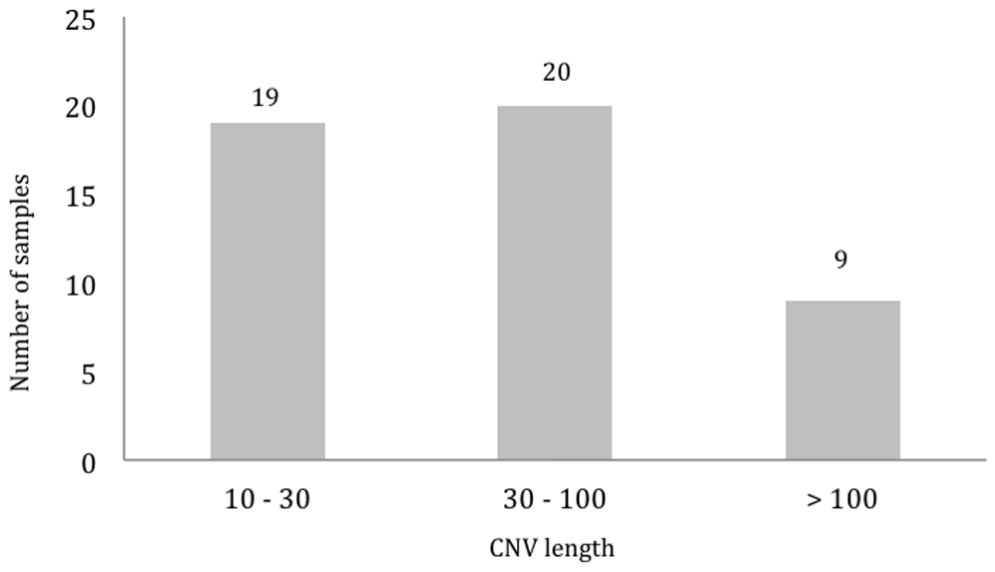
Length distribution of the CNVs unique to the Negrito from Peninsular Malaysia.

Our dataset was further compared with HapMap3 dataset. Analysis revealed 62 CNVs (corresponded to 36 CNVRs) unique to our Negrito samples. However, due to the high false discovery, the CNVs sized <10 kb were excluded from further analysis, hence 48 CNVs remained (24 gains; 24 losses), of which 32 were singletons (Table 4). Length distribution of the CNVRs specific to Negritos is shown in Figure 4.

**Table 4:** Population specific CNVs in 34 genomes of Negrito from Peninsular Malaysia.

Chromosome Cytoband	Start	End	Size	CNV frequency *	CNV type(gain/loss)	Genes involved	Disrupted genes
1p36.22	12,117,038	12,200,251	83,214	0.03	gain	TNFRSF1B, TNFRSF8	TNFRSF8
1q43	237,656,846	237,667,786	10,941	0.03	loss	-	-
2p21	41,716,288	41,781,081	64,794	0.06	loss	-	-
	41,717,149	41,780,408	63,260	0.06	loss	-	-
	41,717,149	41,785,214	68,066	0.03	loss	-	-
2p12	75,457,878	75,486,632	28,755	0.03	gain	-	-
	75,469,904	75,486,632	16,729	0.03	gain	-	-
2q13	109,015,589	109,051,831	36,243	0.03	loss	-	-
2q37.1	232,156,839	232,246,171	89,333	0.03	gain	C2orf57	-
3p26.1	8,096,025	811,9974	23,950	0.03	loss	-	-
3q25.33	161,089,639	161,131,309	41,671	0.03	gain	SCHIP1, IQCJ-SCHIP1	SCHIP1
4q22.2	94,375,625	94,778,350	402,726	0.03	loss	GRID2	GRID2
4q23	100,542,893	100,696,485	153,593	0.03	gain	RG9MTD2, C4orf17, ADH7	RG9MTD2
	100,651,864	100,686,034	34,171	0.03	gain	C4orf17	C4orf17
	100,656,576	100,695,572	38,997	0.03	gain	C4orf17, RG9MTD2	C4orf17, RG9MTD2
4q31.23	149,474,120	149,512,767	38,648	0.03	loss	NR3C2	-
4q32.3	169,960,297	169,996,579	36,283	0.03	loss	PALLD	PALLD
	169,961,956	169,979,040	17,085	0.03	loss	PALLD	-
6p12.2	51,564,000	51,583,519	19,520	0.06	loss	-	-
7p22.2	3,073,605	3,094,449	20,845	0.03	gain	-	-
8p23.2	2,672,753	2,738,233	65,481	0.03	gain	-	-
	2,672,753	2,974,287	301,535	0.03	gain	CSMD1	CSMD1
	2,675,472	2,766,578	91,107	0.03	gain	-	-
	2,780,146	2,947,279	167,134	0.03	gain	CSMD1	CSMD1
8q24.3	143,097,891	143,112,524	14,634	0.03	loss	-	-
9p23	10,734,135	1,0754,097	19,963	0.03	gain	-	-
9p21.1	28,746,344	28,839,949	93,606	0.03	loss	-	-
9q21.33	86,893,365	86,953,896	60,532	0.03	loss	-	-
10q23.1	82,374,114	82,389,577	15,464	0.03	loss	SH2D4B	-
14q13.1	32,721,471	32,746,954	25,484	0.03	loss	NPAS3	-
14q32.12	90,730,514	90,754,443	23,930	0.03	gain	C14orf159	C14orf159
18p11.23, 18p11.31	6,863,354	7,424,329	56,0976	0.03	gain	LRRC30, LAMA1, ARHGAP28, LOC400643	ARHGAP28
18q21.33	58,951,168	58,970,363	19,196	0.03	gain	BCL2	-
19p13.3	4,795,152	4,837,550	42,399	0.09	gain	PLIN3	PLIN3
20p13	2,436,294	2,547,940	111,647	0.03	gain	ZNF343, TMC2	ZNF343, TMC2
	2,436,294	2,555,429	119,136	0.03	gain	ZNF343, TMC2	ZNF343, TMC2
	2,436,294	2,556,157	119,864	0.03	gain	ZNF343, TMC2	ZNF343, TMC2
21q21.2	23,740,931	23,752,189	11,259	0.06	loss	-	-
21q22.3	43,121,093	43,286,636	165,544	0.03	gain	NDUFV3, WDR4, PKNOX1	PKNOX1

Position of CNVs were coordinated based on Human Genome Assembly NCBI (hg18).

*CNV frequencies calculated based on the 34 Negrito genomes genotyped.

To confirm the uniqueness of these CNVs in Negritos, we further compared our dataset with the metropolitan Chinese, Indians and Malays from SGVP. Seven CNVRs were covered in SGVP but none of these putative CNVs we found had been previously reported.

### Gene Ontology and Pathway Analyses

**Figure 5:**
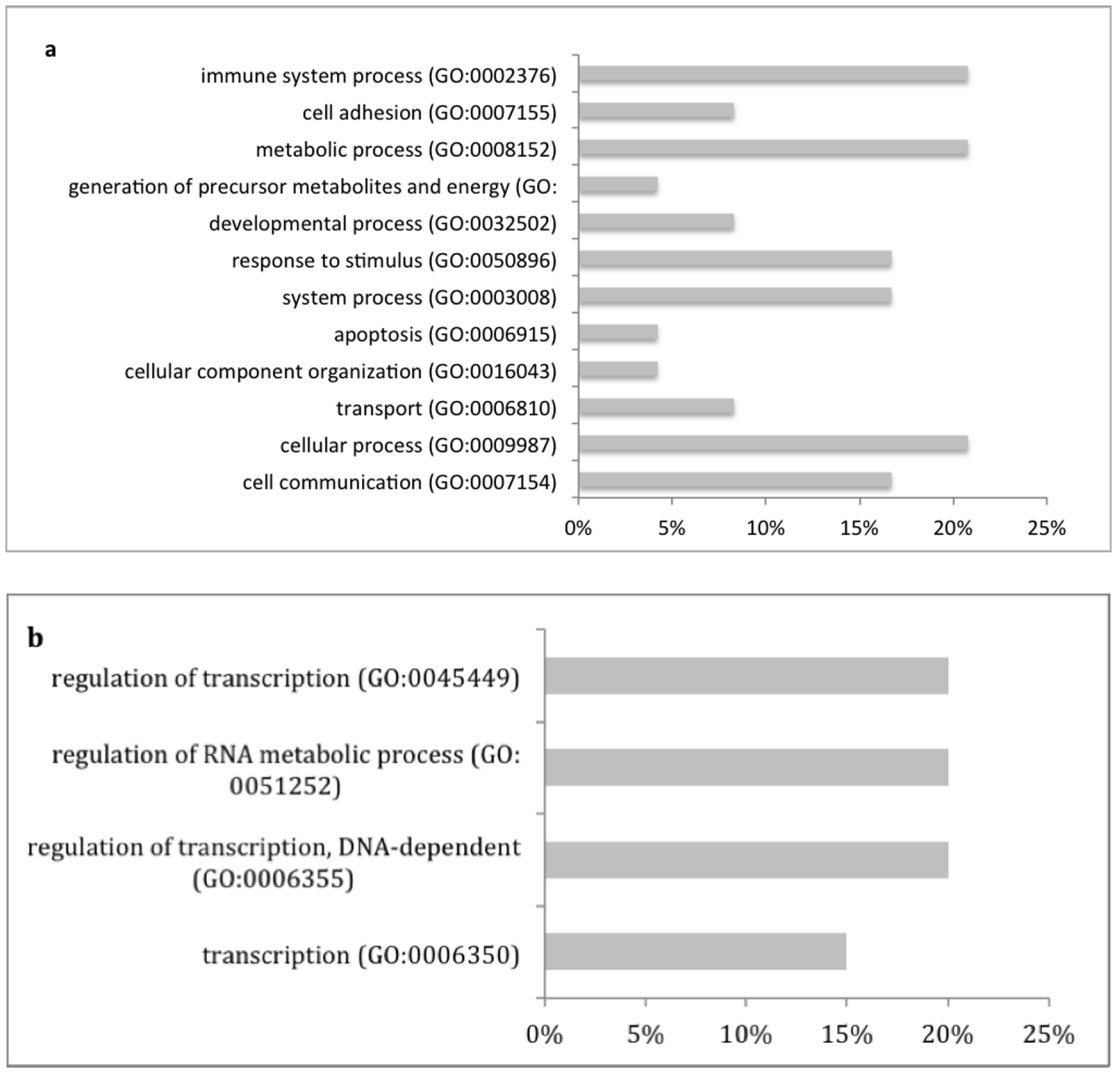
Gene Ontology and pathway analyses on the gene set within the Negrito-specific CNVs using PANTHER and DAVID. (a) PANTHER analysis suggests a major involvement of the genes harboring the population specific CNVs in the immune system process and response to stimulus, as well as the metabolic process; (b) DAVID analysis suggests the involvement of the genes harboring the population specific CNVs in the transcription and regulation of RNA metabolic processes.

To understand the putative functional implications of these CNVs, we performed the Gene Ontology (GO) and pathway analyses on the gene set within the Negrito-specific CNVs using PANTHER and DAVID (Figure 5). Of the 48 CNVs specific to Negritos, 29 carried annotated genes while the remaining were gene-poor regions (Table 4). For all the CNVRs enriched with genes, copy number gains were significantly higher than the losses (15 gain versus 6 losses) (P<0.001). GO analysis by PANTHER revealed fourteen genes involved in immune system function and regulation, response to stimulus and metabolic pathways; whereas DAVID revealed that transcription regulation, and regulation of RNA metabolic processes to be the most significant GO term. The list of genes involved in the major biological processes is listed in Table 5.

**Table 5:** Pathways and biological processes of the genes underlying the population specific CNvs in Negrito from Peninsular Malysia.

Pathways/Biological functions	GO Term	Genes
Immune systems and processes	GO:0002376	TNFRSF8, CSMD1, SH2D4B, TNFRSF1B, LRRC30
Response to stimulus	GO:0050896	TNFRSF8, CSMD1, SH2D4B, TNFRSF1B
System process	GO:0003008	SCHIP1, LAMA1, GRID2, PALLD
Metabolic processes	GO:0008152	NDUFV3, WDR4, NPAS3, ADH7, PKNOX1
Cellular processes	GO:0009987	TNFRSF8, TNFSR1B, LAMA1, GRID2
Cell communication	GO:0007154	TNFRSF8, TNFSR1B, LAMA1, GRID2
Transcription	GO:0006350	NPAS3, NR3C2, ZNF343
Regulation of transcription, DNA-dependent	GO:0006355	PBX1, NPAS3, NR3C2, ZNF343
Regulation of RNA metabolic process	GO:0051252	PBX1, NPAS3, NR3C2, ZNF343

Analysis was performed using PANTHER DAVID.

## Discussion

It is estimated that approximately 96% of the current genome-wide association studies were conducted on individuals of European ancestry [37]. There is a growing need to unveil the spectrum of human genetic diversity by investigating minority populations, for instance the aboriginal populations in Southeast Asia (SEA) countries. The Negrito populations from Peninsular Malaysia are of interest, as they are known to be the descendants of earliest migrants to Southeast Asia. Due to their relatively long period of isolation and semi-nomadic lifestyles, they have had less exposure to urbanization. Their genomes are therefore perceived to be considerably less diverse owing to genetic drift and possibly founder effects. This makes them ideal for investigating genetic forces acting in human evolution, which provides fundamental knowledge to inform disease-based genetic studies as well as gene mapping.

In this study, we identified 263 CNVRs in 34 Negrito subjects from Peninsular Malaysia, of which 27 we believe are novel and unique to Negritos. After excluding the small CNVs, an average 23 CNVs was observed per Negrito genome. It was found to have more losses (72.6%) than gains, in line with most reported studies [12], [29], [32]. Overall size of the CNV observed also corresponded well. Approximately 58% of the CNVs found in Negrito were <30 kb, in line with reports by Yim et al. [27] on the Korean genomes and Ku et al. [32]; but was relatively higher than the reported by Zhang et al. [30] and McElroy et al. [33]. The average number of CNVs detected in the HapMap3 dataset (average CNV call per genome  = 102.2) (data not shown) and the Chinese populations (average CNV call per genome  = 140.9) [29] were much higher. The number of novel CNVRs identified in Negrito was also lower (0.85 per genome) than those previously reported [29]–[30], [32]–[33]. This is expected as we have excluded all the small CNVs <10 kb from our analyses in this study (comprised ∼30.8% of the total CNVs identified). Moreover, more populations being genotyped, the CNV map gets more saturated consequently hence less novel variants are observed. Collectively we observed less CNVRs but more alleles (CNVs) in the Negrito genomes. Though in general, the CNV profile of Negrito genomes looks similar to those reported especially by Ku et al. [32] in three other SEA populations except for the X-chromosome which was not considered in our study.

The variation of the number of CNVs detected could be attributed to several reasons: i) the technology applied for CNV detection and its resolution; ii) levels of stringency applied when performing the CNV call; iii) the algorithms applied when performing CNV call; iv) we excluded the X-chromosome, telomeric and centromeic CNVs. The application of three independent CNV algorithms would minimize the false positive result rates, as evidenced by Pinto et al. [38]. The poor validation rate for the small CNVs (<10 kb) could be attributed to several reasons: (i) poor signal to noise ratio of the samples thus leading to false positive calls by the algorithms; and (ii) inaccurate estimation of breakpoints for the small CNVs due to the limitation of the probe density, thus leading to inaccuracy when identifying a precise CNV during qPCR validation. Therefore precautious should be taken when analysing the small CNVs. Collectively, our approach would increase the confidence of higher quality calls, at an expense of fewer positives being called. Although the number of CNVs was relatively lower then the previous studies, this report is considerably more stringent and with a higher confidence level. We believe more novel CNVRs unique to Negrito could be identified if larger sample sizes were to be investigated.

Interestingly, the CNVRs enriched with genes showed a significantly higher copy number gains. In addition to that, these genes were known to be involved in immunity and response to stimuli, as well as metabolic pathways. We speculate that the Negrito may have undergone processes of local adaptation and positive selection, which necessitated their expansions and eventual settlement in forest habitats. This hypothesis is supported by several previously reported studies [24], [30], [39]. However, possibilities of other processes such genetic drift due to random duplications or deletions should not be ruled out [40]. Nevertheless, further investigations should be carried out to confirm the findings.

The health of Negritos has not been studied comprehensively for several decades and there are few recent publications [9]. However, early studies indicated that Negritos were under various medical stresses especially with high prevalence of communicable diseases including malaria, tuberculosis, leptospirosis and various intestinal infections [41]. This could be attributed to their life style in the early days, whereby the hunting-gathering activities were practiced hence are exposed to a variety of transmissible diseases. Malnutrition has been reported to be common amongst aboriginal communities, especially women [42]. Although there are no specific reports on the nutritional status on Negritos of late, our observations and direct communications with the Negrito tribes lead us to believe that the majority is undernourished. Although we cannot provide unequivocal evidence, it is conceivable that their biomedical stresses experienced in over the years resulted in the enrichment of selected genes in these Negrito specific CNVs.

This is the first study of genome-wide CNVs in the Negrito population from Peninsular Malaysia. We identified putative novel CNVs unique to the Negrito populations from Peninsular Malaysia. Although the smaller sample size does not allow us to perform functional and statistical analysis, our data was analyzed with most stringent QC criteria and were then compared with a number of datasets including DGV, HapMap3 and the SGVP. As such, we think our data is highly reliable.

Population studies to catalogue the patterns, frequencies and distribution of CNV in non-disease based cohort is crucial to provide fundamental understanding of its impact to the phenotypic diversity and disease susceptibility. Hence, characterization of more diverse populations is needed to improve the saturation of CNV map for human genome. To this end, we are continuing our investigations amongst the indigenous in Malaysia.

## Summary

Our findings provide fundamental knowledge, different perspectives and insights to the genetic diversity of Negritos of Peninsular Malaysia. This can inform studies of local adaptation, natural selection and also potentially influence health programmes in the near future.

## References

[1] Li J, Seielstad M, Xiao C (2001) Genetic, linguistic and archeological perspectives on human diversity in Southeast Asia. World Scientific Publishing. Singapore.

[2] Barker G, Barton H, Bird M, Daly P, Datan I, et al (2007) The ‘human revolution’ in lowland tropical Southeast Asia: The antiquity and behavior of anatomically modern humans at Niah Cave (Sarawak, Borneo). J Hum Evol 52: 243–261. 17161859 10.1016/j.jhevol.2006.08.011

[3] Stringer CB, Andrews P (1998) Genetic and fossil evidence for the origin of modern humans. Science 239: 1263–1268. 10.1126/science.3125610 3125610

[4] Macaulay V, Hill C, Achilli A, Rengo C, Clarke D, et al (2005) Single, rapid coastal settlement of Asia revealed by analysis of complete mitochondrial genomes. Science 308: 1034–1036. 15890885 10.1126/science.1109792

[5] Lahr MM, Foley R (1994) Multiple dispersals and modern human origins. Evol Anthro 3: 48–60.

[6] The HUGO Pan-Asian SNP Consortium (2009) Mapping human genetic diversity in Asia. Science 326: 1541–1545. 20007900 10.1126/science.1177074

[7] Bellwood P (1993) Cultural and biological differentiation in Peninsular Malaysia: The last 10,000 years. Asian Perspec 32: 37–60.

[8] Hill C, Soares P, Mormina M, Macaulay V, Meehan W, et al (2006) Phylogeography and ethnogenesis of aboriginal Southeast Asians. Mol Biol Evol 23: 2480–2491. 16982817 10.1093/molbev/msl124

[9] Jinam TA, Phipps ME, Indran M, Kuppusamy UR, Mahmood AA, et al (2008) An update of the general health status in the indigenous populations of Malaysia. Ethn Health 13: 277–287. 18568977 10.1080/13557850801930478

[10] Hoh BP, Nur Shafawati AR, Yam YY (2011) Characterization of seven (CA)_n_ markers among three populations of negritos from Peninsular Malaysia. Malays Appl Biol 40: 61–67.

[11] Peng MS, Quang HH, Dang KP, Trieu AV, Wang HW, et al (2010) Tracing the Austronesian footprint in mainland Southeast Asia: perspective from mitochondrial DNA. Mol Biol Evol 27: 2417–2430. 20513740 10.1093/molbev/msq131

[12] Kristiansson K, Naukkarinen J, Peltonen L (2010) Isolated populations and complex disease gene identification. Genome Biol 9: 109. 10.1186/gb-2008-9-8-109 PMC2575505 18771588

[13] Redon R, Ishikawa S, Fitch KR, Feuk L, Perry GH, et al (2006) Global variation in copy number in the human genome. Nature 444: 444–454. 17122850 10.1038/nature05329 PMC2669898

[14] Girirajan S, Campbell CD, Eichler EE (2011) Human copy number variation and complex genetic disease. Annu Rev Genet 45: 203–226. 21854229 10.1146/annurev-genet-102209-163544 PMC6662611

[15] Gonzalez E, Kulkarni H, Bolivar H, Mangano A, Sanchez R, et al (2005) The influence of CCL3L1 gene-containing segmental duplications on HIV-1/AIDS susceptibility. Science 307: 1434–1440. 15637236 10.1126/science.1101160

[16] Pinto D, Pagnamenta AT, Klei L, Anney R, Merico D, et al (2010) Functional impact of global rare copy number variation in autism spectrum disorders. Nature 466: 368–372. 20531469 10.1038/nature09146 PMC3021798

[17] Hollox EJ, Huffmeier U, Zeeuwen PL, Palla R, Las-corz J, et al (2007) Psoriasis is associated with increased beta-defensin genomic copy number. Nat Genet 40: 23–25. 18059266 10.1038/ng.2007.48 PMC2447885

[18] Stranger BE, Forrest MS, Dunning M, Ingle CE, Beazley C, et al (2007) Relative impact of nucleotide and copy number variation of gene expression phenotypes. Science 315: 848–853. 17289997 10.1126/science.1136678 PMC2665772

[19] Henrichsen CN, Chaignat E, Reymond A (2009) Copy number variants, diseases and gene expression. Hum Mol Genet 18: R1–8. 19297395 10.1093/hmg/ddp011

[20] Estivill X, Armengol L (2007) Copy number variants and common disorders: Filling the gaps and exploring complexity in genome-wide association studies. PLoS Genet 3: 1787–1799. 17953491 10.1371/journal.pgen.0030190 PMC2039766

[21] Korbel JO, Kim PM, Chen X, Urban AU, Weissman S, et al (2008) The current excitement about copy-number variation: How it relates to gene duplication and protein families. Curr Opin Struc Biol 18: 366–374. 10.1016/j.sbi.2008.02.005 PMC2577873 18511261

[22] Itsara A, Wu H, Smith JD, Nickerson DA, Romieu I, et al (2010) De novo rates and selection of large copy number variation. Genome Res 20: 1469–1481. 20841430 10.1101/gr.107680.110 PMC2963811

[23] Cooper GM, Nickerson DA, Eichler EE (2007) Mutational and selective effects on copy-number variants in the human genome. Nat Genet 39: S22–S29. 17597777 10.1038/ng2054

[24] Nguyen DQ, Webber C, Ponting CP (2006) Bias of selection on human copy-number variants. PLoS Genet 2: e20. 16482228 10.1371/journal.pgen.0020020 PMC1366494

[25] The International Haplotype Map Consortium (2005) A haplotype map of the human genome. Nature 437: 1299–1320. 16255080 10.1038/nature04226 PMC1880871

[26] Li JZ, Absher DM, Tang H, Southwick AM, Casto AM, et al. (2008) Worldwide human relationships inferred from genome-wide patterns of variation. Science 319: , 1100–1104. 10.1126/science.1153717 18292342

[27] Yim SH, Kim TM, Hu HJ, Kim JH, Kim BJ, et al (2010) Copy number variations in East-Asian population and their evolutionary and functional implications. Hum Mol Genet 19: 1001–1008. 20026555 10.1093/hmg/ddp564 PMC2830825

[28] Gautam P, Jha P, Kumar D, Tyagi S, Varma B, et al (2012) Spectrum of large copy number variations in 26 diverse Indian populations: potential involvement in phenotypic diversity. Hum Genet 131: 131–143. 21744140 10.1007/s00439-011-1050-5

[29] Lou H, Li S, Yang Y, Kang L, Zhang X, et al (2011) A Map of copy number variations in Chinese populations. PLoS One 6: e27341. 22087296 10.1371/journal.pone.0027341 PMC3210162

[30] Zhang YB, Li X, Zhang F, Wang DM, Yu J (2012) A preliminary study of copy number variation in Tibetans. PLoS One 7: e41768. 22844521 10.1371/journal.pone.0041768 PMC3402393

[31] Chen W, Hayward C, Wright AF, Hicks AA, Vitart V, et al (2011) Copy number variation across European populations. PLoS One 6: e23087. 21829696 10.1371/journal.pone.0023087 PMC3150386

[32] Ku CS, Pawitan YP, Sim X, Ong RT, Seielstad M, et al (2010) Genomic copy number variation in three Southeast Asian Populations. Hum Mutat 31: 851–857. 20506136 10.1002/humu.21287

[33] McElroy JP, Nelson MR, Caillier SJ, Oksenberg JR (2009) Copy number variation in African Americans. BMC Genet 10: 15. 19317893 10.1186/1471-2156-10-15 PMC2674062

[34] Lionel AC, Crosbie J, Barbosa N, Goodale T, Thiruvahindrapuram B, et al (2011) Rare Copy Number Variation Discovery and Cross- Disorder Comparisons Identify Risk Genes for ADHD. Sci Trans Med 3: 95ra75. 10.1126/scitranslmed.3002464 21832240

[35] Stephens SH, Logel J, Barton A, Franks A, Schultz J, et al (2009) Association of the 5'-upstream regulatory region of the alpha7 nicotinic acetylcholine receptor subunit gene (CHRNA7) with schizophrenia. Schizophr Res 109: 102–12. 19181484 10.1016/j.schres.2008.12.017 PMC2748327

[36] Taske NL, Williamson MP, Makoff A, Bate L, Curtis D, et al (2002) Evaluation of the positional candidate gene CHRNA7 at the juvenile myoclonic epilepsy locus (EJM2) on chromosome 15q13-14. Epilepsy Res 49: 157–72. 12049804 10.1016/s0920-1211(02)00027-x

[37] Bustamante CD, Burchard EG, De La Vega FM (2011) Genomics for the world. Nature 475: 163–165. 21753830 10.1038/475163a PMC3708540

[38] Pinto D, Darvishi K, Shi X, Rajan D, Rigler D, et al (2011) Comprehensive assessment of array-based platforms and calling algorithms for detection of copy number variants. Nat Biotechnol 29: 512–520. 21552272 10.1038/nbt.1852 PMC3270583

[39] Otto SP, Yong P (2002) The evolution of gene duplicates. 46: 451–483. 10.1016/s0065-2660(02)46017-8 11931235

[40] Nozawa M, Kawahara Y, Nei M (2007) Genomic drift and copy number variation of sensory receptor genes in humans. Proc Natl Acad Sci 104: 20421–20426. 18077390 10.1073/pnas.0709956104 PMC2154446

[41] Baer A (1999) Health, disease and survival: a biomedical and genetic analysis of the Orang Asli of Malaysia. Center for Orang Asli Concerns. Kuala Lumpur, Malaysia.

[42] Osman A, Zaleha MI (1995) Nutritional status of women and children in Malaysian rural populations. Asian Pac J Clin Nutr 4: 319–324. 24394359

